# Augmented Reality Exergames for Upcoming Cognitive-Motor Rehabilitation: User-Centered Design Approach and User Experience of Healthy Children

**DOI:** 10.2196/69205

**Published:** 2025-02-19

**Authors:** Maxime Balloufaud, Arnaud Boujut, Romain Marie, Aurélie Guinaldo, Laurent Fourcade, Julia Hamonet-Torny, Anaick Perrochon

**Affiliations:** 1 Univ.Limoges HAVAE UR 20217 F-87000 Limoges France; 2 3iL Ingénieurs Limoges France; 3 Pediatric Surgery Division University Hospital CHU de Limoges Limoges France; 4 Centre Hospitalier Esquirol Limoges Limoges France

**Keywords:** augmented reality, exergames, user experience, healthy children, cognitive-motor intervention

## Abstract

**Background:**

Traditional rehabilitation programs for children with cerebral palsy and acquired brain injuries aim to enhance motor and cognitive abilities through repetitive exercises, which are often perceived as tedious and demotivating. Extended reality technologies, including augmented reality (AR) and virtual reality, offer more engaging methods through exergames. However, to date, no AR exergames simultaneously integrate cognitive and motor aspects within navigational tasks. Developing these exergames necessitates rigorous methodological steps, especially when using emerging technologies such as AR. The MIDE (Multidisciplinary Iterative Design of Exergames) framework advocates a participatory design approach, involving users from the outset, the objective being to optimize the interface and validate game mechanics through user experience (UX) assessment. Some researchers initially test these mechanisms on healthy children before applying them to clinical populations.

**Objective:**

This study aims to evaluate the UX of our AR exergames, consisting of two games (AR Corsi and AR Zoo), in typically developing children.

**Methods:**

Typically developing children participated in two 1.5-hour sessions. During each session, they played one of two AR games using the Microsoft HoloLens 2 headset: AR Corsi and AR Zoo, both of which are designed to engage executive functions and motor skills through navigational capabilities. UX was assessed after each session using the following measures: System Usability Scale scores for usability, AttrakDiff for attractiveness and game quality, MeCue for emotional experience, and Rating scale of Perceived Exertion for Children for pre- and postsession mental and physical fatigue.

**Results:**

A total of 27 participants (mean age 11.9, SD 1.2 years) were included in the study. Mean System Usability Scale scores were 79.9 (SD 11.4) for AR Corsi and 76.3 (SD 12.1) for AR Zoo, indicating good usability. The AttrakDiff questionnaire yielded favorable results, with scores between 1 and 3 for overall attractiveness, pragmatic quality, and stimulation for both AR games. However, the hedonic quality “identity” received neutral scores (mean 0.6, SD 0.5 for AR Corsi and mean 0.7, SD 0.8 for AR Zoo). The MeCue emotions module yielded average scores of 5.2 (SD 0.7) for AR Corsi and 5.3 (SD 0.8) for AR Zoo, significantly exceeding the theoretical mean of 4 (*P*<.001). We observed a significant effect of physical fatigue (*P*=.02) and mental fatigue (*P*=.002) after exposure to both games. A comparative analysis of UX between the two games showed no significant differences.

**Conclusions:**

This study demonstrates that our exergame, comprising two AR games, is user-friendly and well-received by typically developing children, eliciting positive emotions and overall appeal. Although some children reported fatigue, favorable UX evaluation confirms the validity of the game’s content and mechanisms, suggesting its suitability for use among children with cerebral palsy and acquired brain injuries.

## Introduction

Traditional rehabilitation programs for children with cerebral palsy (CP) and acquired brain injury (ABI) primarily aim to enhance motor abilities, such as cognition and visuospatial exploration, through repetitive exercises [1,2]. However, these exercises are often perceived as boring, tedious, and demotivating, reducing their effectiveness and leading to low adherence [3,4]. It is essential to explore more engaging and appealing approaches designed to sustain motivation throughout the rehabilitation process. That said, these programs also present additional challenges, particularly in simultaneously addressing motor aspects, such as walking, and cognitive aspects, such as executive functions, while incorporating spatial navigation tasks, which are frequently impaired in children with CP and ABI [5,6].

In this context, “extended reality” (XR) systems, which include both virtual reality (VR) and augmented reality (AR) technologies [7,8], have demonstrated effectiveness and positively impacted therapy adherence and motivation in young patients with CP and ABI [9-11]. More specifically, XR devices that combine games and exercises, commonly referred to as “exergames,” provide a more playful and immersive alternative to traditional rehabilitation programs [12]. These technologies enable children to perform complex movements in a safe and immersive virtual environment [13], thereby enhancing their engagement, learning, and self-confidence while making the rehabilitation process more enjoyable [14].

Although systematic reviews and meta-analyses have confirmed the effectiveness of VR in motor rehabilitation for young patients with CP and ABI [9,10], studies specifically examining the use of immersive AR in these children remain limited. To date, research in AR has primarily focused on gait training [15,16], without integrating cognitive components or spatial navigation, which are critical for these populations. However, AR offers several significant advantages, including a reduction in side effects such as cybersickness [17], which is commonly associated with VR, while maintaining a high level of immersion and interaction [8,18]. Moreover, AR provides greater flexibility in designing spatial navigation exercises by leveraging the integration of virtual elements into the child’s actual environment. Developing AR-based cognitive-motor exergames could offer an innovative and engaging approach for young patients, addressing both their physical and cognitive stimulation needs.

The development of an XR rehabilitation device is complex and involves multiple methodological steps [12,19,20] to ensure usability, acceptability, and effectiveness. Among current methodologies, the MIDE (“Multidisciplinary Iterative Design of Exergames”) framework applies a multidisciplinary approach and an iterative methodology to ensure that each exergame meets necessary health objectives while providing an appealing and accessible design for its target demographic [21]. This framework comprises a three-phase approach: (1) contextual research is conducted to understand the needs of users and therapists through direct interactions and literature review; (2) the design and development phase entails collaboration between therapists and users to cocreate and customize game content; and (3) the device’s effectiveness, usability, and user acceptance are evaluated. Even with guidelines, only 10% of studies incorporate user experience (UX) evaluation in the design of rehabilitation video games for children [22].

A comprehensive understanding of UX is crucial when developing a novel rehabilitation device. User-centered models facilitate deeper comprehension of the needs and expectations of the users for whom these systems are created. For instance, Hassenzahl [23] proposes a theoretical model of UX that distinguishes between the perspectives of the designer and the user, where the designer implements elements such as content and functionality to foster positive pragmatic and hedonic qualities in the user, thereby significantly influencing the user’s overall perception of the device’s attractiveness. Similarly, Mahlke [24] includes emotions as an important component in his model, acknowledging their vital role in the UX. Emotions directly affect perceptions of usefulness, usability, and other noninstrumental qualities such as esthetics, highlighting the need to take them into account in the design of digital systems. Consequently, assessing the UX of an XR headset will encompass several aspects, including usability, ease of learning, satisfaction, emotions, and other contributory factors.

UX evaluation is essential to the development of innovative technological tools assessing the feasibility of a project, particularly when the user is placed at the center of development [14,25]. By involving users early in the developmental process, it becomes possible to better address their specific needs and promote the adoption of the technology [12,25,26], especially when it is new, one example being AR headsets. User involvement through a participatory design approach, as demonstrated by Eisapour et al [27], facilitates the creation of an exergame that aligns with user preferences while identifying and resolving potential hidden issues. In this study [27], the design process relied on several iterative cycles of testing and revisions based on user feedback, ensuring user-centered development that would optimize the final product’s quality and effectiveness.

To enhance this participatory approach and ensure successful adoption, some researchers begin by evaluating healthy participants, the objective is to validate the game’s mechanisms and optimize its design before introducing it to clinical populations [28,29]. Indeed, pilot and feasibility studies establish a foundation for future randomized controlled trials involving pathological participants. Free from the confounding effects introduced by pathological variables, the preliminary phase validates the underlying concepts, mechanisms, and design of the game. This approach enables designers to confirm that both technically and conceptually, the game functions as intended. Additionally, testing the game on healthy participants establishes a performance baseline, which is crucial to the assessment of the game’s actual impact on patients in later stages of development.

This study aims to evaluate UX in terms of usability, emotions, and fatigue in an AR exergame composed of two games targeting typically developing children. Additionally, this study seeks to determine whether there are significant differences between the two AR games in UX outcomes. Our primary hypothesis is that the AR exergame will be easily usable by children, thereby underlining the feasibility of conducting cognitive-motor exercises in AR for this population. We expect the overall UX to be positive. Furthermore, we hypothesize that AR-based sessions will not result in significantly increased physical or mental fatigue. Regarding the secondary hypothesis, we do not anticipate any significant differences between the two AR games.

## Methods

### Study Design

We recruited children and adolescents from leisure centers in the Haute-Vienne department (France). The inclusion criteria were as follows: children aged between 10 and 16 years with typical development (no diagnosis of developmental disorders, developmental delays, or mental or physical health problems) and sufficient oral comprehension skills. The noninclusion criteria were: children with locomotor disabilities (eg, casts and crutches), contraindications for using XR technologies (eg, photosensitive epilepsy), atypical or uncorrected visual or hearing impairments, unstable medical conditions, or refusal by the child or family, which would prevent inclusion.

### Ethical Considerations

This study followed the principles of the Declaration of Helsinki and obtained approval from the ethics commission (IRB00012476-2023-18-10-273). Assent from the child and written informed consent from a legal representative were obtained. Additionally, all participant data were anonymized.

### Protocol

Following the MIDE framework proposed by Li et al [21], we used a multidisciplinary approach and an iterative methodology to ensure that our exergame would meet the necessary health objectives while maintaining an attractive and accessible design for its target demographic.

### Step 1 of MIDE: Contextual Research

We first established clear objectives for the development of an exergame targeting cognitive-motor rehabilitation of children and adolescents with CP or ABI. This initial phase was grounded in the combined expertise of the project team members and a thorough review of the scientific literature. Rehabilitation needs specific to these young individuals were central to the game’s design. To optimize the rehabilitation of motor and cognitive functions, the tasks we designed need to be repetitive, progressive, goal-oriented, and tailored to each individual [30-32], and it is also crucial that the rehabilitation we offer both motivate and engage through gamification. Furthermore, it is necessary to enhance motor and cognitive skills through spatial navigation tasks involving executive functions [5,33].

To ensure the success of this development, we assembled a multidisciplinary team that included specialists in CP and ABI (doctors of physical and rehabilitation medicine), game developers, human-computer interaction experts, XR technology researchers, and UX specialists. Each team member was assigned specific roles based on their expertise, covering all aspects of the development process, from game mechanics to user interface design. For this development, we leveraged our previous experiences with digital technology for cognitive-motor rehabilitation.

In earlier work within our laboratory, we used virtual carpet technology, a projection system, described in an experimental study [34] to develop an exergame for cognitive-motor rehabilitation in older individuals [35]. This prior 2D setup proved cumbersome to implement, requiring various trackers to detect user movements. Building on these findings, we opted to explore an AR system in order to simplify the setup by incorporating motion sensors (ie, accelerometer, gyroscope, and magnetometer) directly into the headset. This approach allows for the design of mobility and interaction scenarios between the real world and 3D holograms.

### Step 2 of MIDE: Design and Development of a New AR Exergame

#### Hardware for AR Exergame

In our exergame, we used the Microsoft HoloLens 2 AR headset, which enables virtual objects to be overlaid in the real environment. This headset indicates the position coordinates (x, y, z) of the participant, allowing for six mechanical degrees of freedom of movement and interaction with the surrounding 3D space. It is portable and lightweight, and due to the seamless integration of holograms, it does not obstruct the user’s view of the real world. HoloLens 2 features a resolution of 2048×1080 per eye, a refresh rate of up to 75 Hz, and a 52° diagonal field of view.

**Figure 1:**
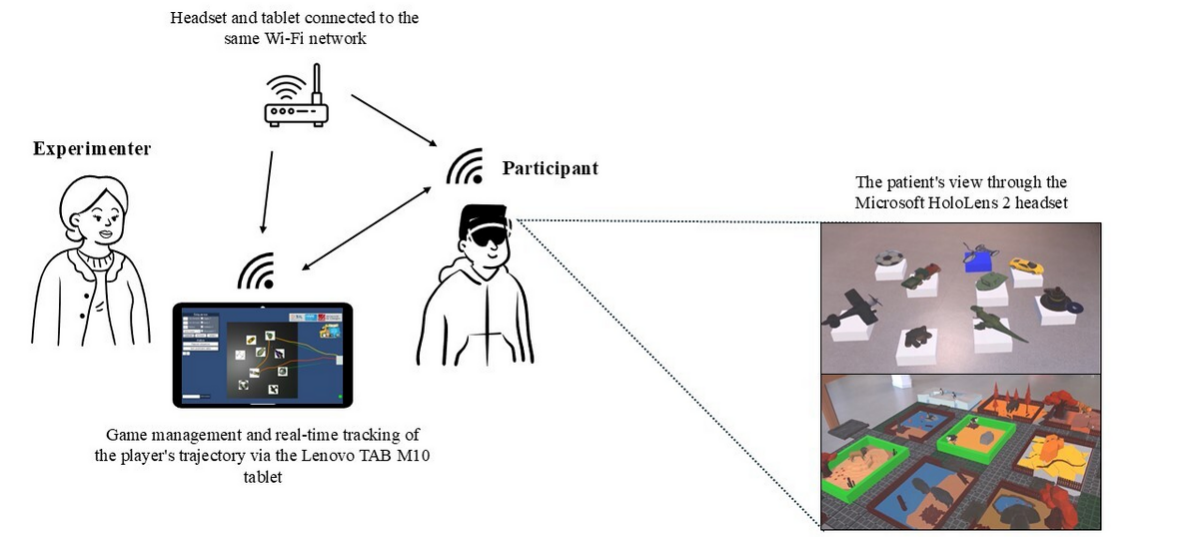
Representation of system interactions.

To enhance UX, particularly for children, our device is designed in the “Plug and Play” mode. Game management, including condition settings and difficulty levels, is controlled by a therapist using a Lenovo TAB M10 Plus tablet. This tablet runs on the Android 13 operating system and boasts a display resolution of 2000×1200. Communication between the two devices is established via Wi-Fi using an IP4 address. Figure 1 illustrates the interaction between the components of our system, ensuring the instantaneous launching of the game once the connection is established.

#### AR System Software

The AR system (version 2021.3.26f1) and tablet (version 2019.4.21f1) applications were developed using Unity (Unity Technologies Corp) software, using various Unity assets to enhance the UX with key visual and interactive elements for pediatric rehabilitation. Visual Studio (Microsoft Corp) was used for application development and coding. Due to the rapid expansion of the XR headset market in recent years, we have designed our games to be deployable on similar HMD devices, requiring only minimal technical adjustments.

We developed two AR games with innovative variations, one of them entailing the integration of a virtual agent based on the motor learning model [31]. As recommended for this model, these games incorporate a large number of repetitions of functional tasks, visual and auditory feedback, and a gradual increase in task difficulty, all of which are essential to the rehabilitation of children with CP or ABI.

#### AR Games

##### AR Corsi Game

This game draws inspiration from the Magic Carpet (MC) [36-39] and the Virtual Walking Corsi Test [34], both of which are adaptations of the Walking Corsi test [40] for locomotor space. The AR Corsi game was developed to address issues of spatial orientation, visuospatial memory, and cognitive strategies to be deployed during navigation. These tests transpose the Corsi block-tapping task [41] into a format where participants navigate on tiles instead of tapping on cubes. It is one of the most widely used neuropsychological tests assessing visuospatial memory. In this test, participants memorize sequences of increasing length by tapping on small wooden or plastic cubes. By contrast, the MC or Virtual Carpet presents sequences in locomotor space using an electronic device, requiring participants to retrieve them by walking on physical or virtual tiles. The AR Corsi game maintains the same dimensions as the Virtual Walking Corsi Test, featuring nine 3D white square tiles (30 cm×30 cm) arranged in a geometric layout like that of the Corsi block-tapping blocks on a virtual board measuring 300 cm×240 cm. Positioned outside the board, the 10th tile serves as the starting and finishing point. The difficulty levels range from 2 to 9 tiles to navigate.

**Figure 2:**
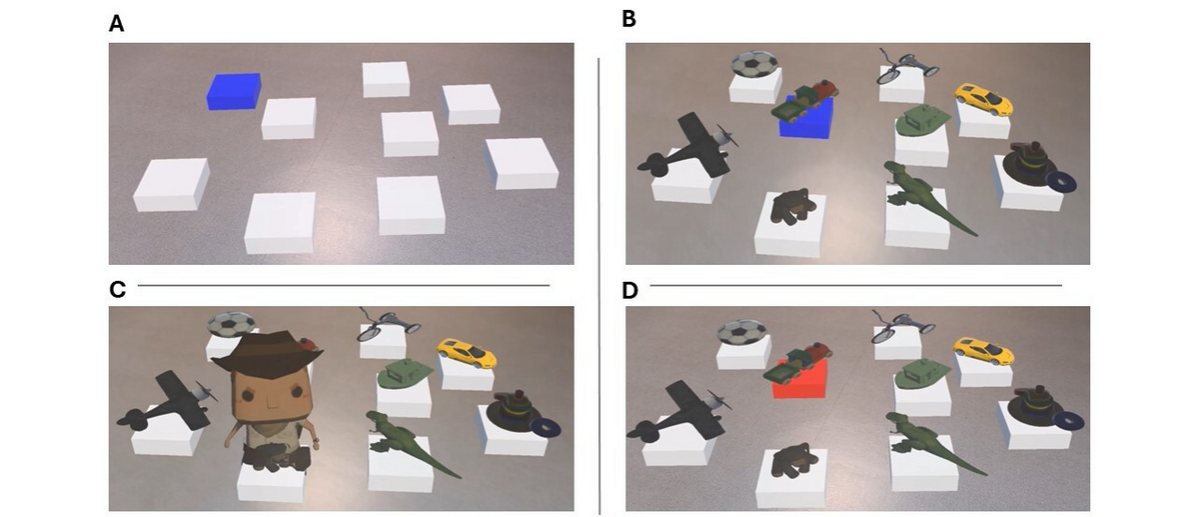
AR Corsi game conditions. (A) Condition “Classic.” (B) Condition “Classic with objects.” (C) Condition “Virtual Agent.” (D) Condition “Advanced.”.

The AR Corsi game offers four distinct and novelty conditions (Figure 2 and Multimedia Appendix 1).

- The “Classic” condition (Figure 2A) entails memorizing a sequence of virtual tiles that are illuminated in blue. Players must then reproduce the sequence by navigating the real environment. A sound cue signals the start of the sequence, after which players can commence at their own pace. While navigating, players validate each tile by remaining on it for one second, causing it to illuminate in green and triggering the validation sound signal. Upon completing the sequence, players return to the starting tile.
- The “Classic with objects” (Figure 2B) condition is akin to the first condition but incorporates objects on the tiles. Objectives and instructions remain unchanged, with auditory feedback reproducing the sounds of the objects during the memorization phase. Additionally, spatial cues such as objects will enable participants to develop alternative cognitive strategies for task resolution, as they will be able to use language as well [42].
- In the “Virtual Agent” condition, a virtual agent depicted as an explorer presents the sequence to the player. The virtual agent traverses from one object to another every second, requiring players to memorize its path and reproduce it in accordance with the criteria of the first two conditions. We hypothesize that the integration of this virtual agent will enhance spatial memory and improve player motivation by making the experience more engaging (Figure 2C).
- The final condition, “Advanced,” tasks players with memorizing the order of objects illuminated in blue and inhibiting those illuminated in red. Instructions mirror those of the other conditions (Figure 2D). This condition is more challenging, as it is likely to engage additional executive functions, such as inhibition and cognitive flexibility, in addition to working memory.

##### AR Zoo Game

The AR Zoo game is an adaptation of the Zoo Map Test, a commonly used assessment tool evaluating executive functions in children, as part of the Behavioral Assessment of the Dysexecutive Syndrome for Children battery [43-45]. In the traditional neuropsychological test, participants are presented with a map of a zoo on a sheet of paper and tasked with planning a route to visit several animals, adhering to various constraints such as avoiding the retracing of certain paths and selecting the fastest route. The test comprises two conditions: the first emphasizes planning skills, requiring participants to anticipate the order of visiting designated locations in view of minimizing errors, while the second condition is less demanding, simply instructing participants to follow directions in view of completing an error-free journey.

The AR Zoo game aims to address challenges related to spatial orientation, planning, and cognitive strategies to be applied during navigation. We have adapted the fundamental concept by modifying aspects related to the animals, zoo layout, and game conditions while introducing spatial navigation. To enhance accessibility for children, we selected a zoo with 12 easily identifiable animated 3D animal enclosures. The game board measures 500 cm×410 cm, with enclosures measuring 80 cm×75 cm and paths 30 cm wide. Each enclosure is equipped with a validation slab measuring 30 cm×30 cm. The difficulty levels range from two to six animals.

**Figure 3:**
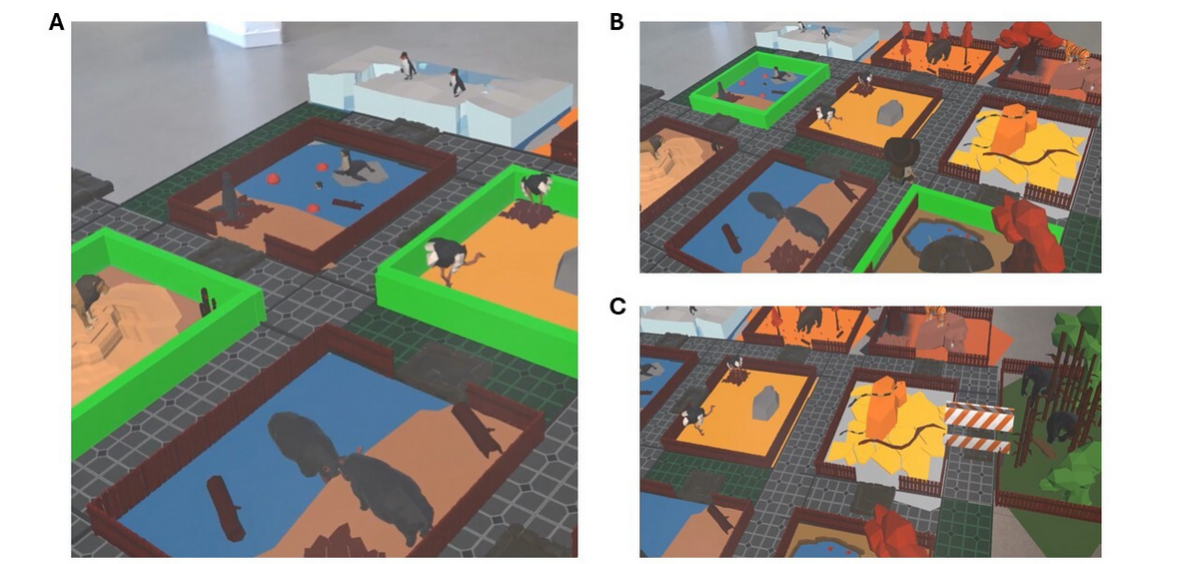
AR Zoo game conditions. (A) Condition “Classic.” (B) Condition “Virtual Agent.” (C) Condition “Advanced.”.

AR Zoo offers three distinct conditions (Figure 3 and Multimedia Appendix 2):

- In the first condition, known as “Classic,” the player commences on the starting slab and visits the animal enclosures in a chosen order while adhering to specific instructions. They must navigate the quickest route, beginning at the starting slab and concluding at the exit slab. Grey paths are for single use, while green paths can be taken multiple times. To validate a visit to an animal enclosure, the player must remain on the tile in front of the corresponding enclosure for one second (indicated by a green light and the sound of the animal). When the therapist initiates a sequence, the enclosures of the animals to be visited are illuminated in green. Players can take their time planning their itinerary before departing from the starting tile to complete the sequence. Once the player has left the starting tile, the enclosures to visit are no longer highlighted in green, thereby introducing a memory component to the condition. Once the sequence is completed, the player must validate it by ending at the exit slab.
- The second condition, termed “Virtual Agent,” involves a virtual agent demonstrating the optimal route for the player to follow, while adhering to the instructions provided in the “Classic” condition. Positioned on the starting tile, the player observes the virtual explorer navigating the journey. Once the virtual agent reaches the exit slab, the player can commence their journey, attempting to replicate the same route according to the previously stated instructions. Similar to the AR Corsi game, we hypothesize that integration of the virtual agent will facilitate learning by showing the optimal path to the player. This approach may potentially reduce the child’s cognitive load, allowing them to focus not on planning but rather on visuospatial memory.
- The third condition, designated as “Advanced,” focuses on learning the optimal path. Initially, the player follows a sequence as the virtual agent displays the optimal route, which is akin to the “Virtual Agent” condition. Subsequently, similar to the “Classic” condition, the player repeats the same sequence independently. Finally, the player is requested to devise a new route based on memorized information, as obstacles appear on the fastest route following departure from the starting slab. Three obstacles appear simultaneously, and cannot be bypassed by the player. Instructions mirror those of the first two conditions. This condition is more challenging due to the requirement for the player to not only memorize the optimal path but also to demonstrate cognitive flexibility and replanning skills enabling them to navigate around the obstacles having emerged along the route during execution of this type of task [46].

#### Therapist Interface

Supervision of game progress is essential to ensuring an optimal gaming experience. This involves adjusting difficulty levels, providing assistance, and managing game conditions according to the player’s needs and abilities. For instance, therapists can intervene by replaying a sequence that a child struggles to recall, thereby sustaining motivation and engagement.

**Figure 4:**
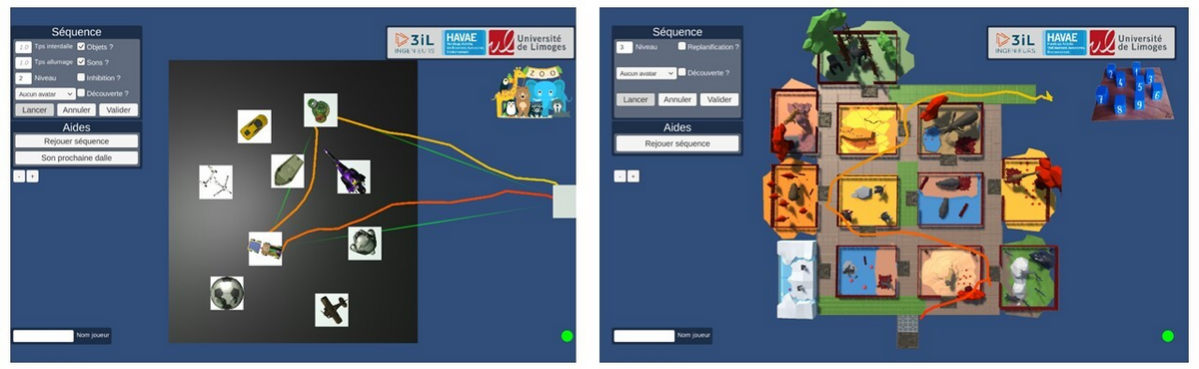
Tablet interface and traced player trajectory.

Access to specific in-game controls, such as manually starting, validating, or canceling game sequences, offers therapists the flexibility to adapt sessions to unforeseen circumstances. As depicted in Figure 4, trajectory tracking provides therapists with a valuable visual aid for real-time performance assessment and player feedback. This is made possible by real-time tracking of the headset’s x, y, and z trajectory coordinates.

Switching between games is seamless; therapists need only tap the logo at the top right corner of the tablet. In fact, a unified application has been developed, consolidating AR Corsi and AR Zoo games.

### Step 3 of MIDE: System Evaluation

#### Overview

**Figure 5:**
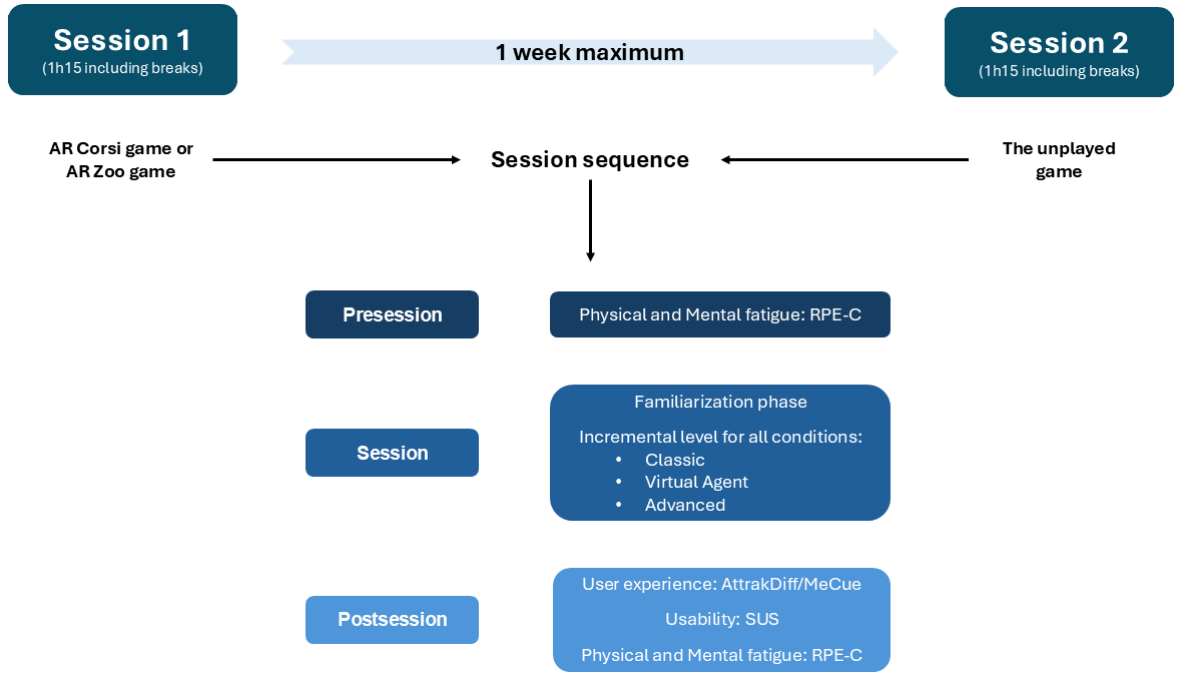
The study procedure. RPE-C: Rating scale of Perceived Exertion for Children; SUS: System Usability Scale.

The two-session UX study procedure is displayed in Figure 5. The second session was scheduled no later than one week after the initial session and mirrored its structure. The order of the games was counterbalanced. Each session comprised a familiarization phase and a gameplay phase. The first aimed to acclimate players to the technology, to familiarize them with navigating the AR environment, and to help them comprehend the instructions for each condition. During this phase, players freely explored the virtual elements (objects or animals) and completed an initial “white” sequence of minimal difficulty, accompanied by real-time guidance and instructions from the therapist. The duration of the familiarization period varied according to the child’s comprehension level. The actual condition commenced once the player had successfully validated two sequences consecutively at the minimum difficulty level.

Following this phase, the gameplay phase entailed execution of the different conditions with escalating difficulty levels, starting at the minimum level and progressing to the player-specific maximum level. A child could advance to the next difficulty level upon having successfully completed at least one of the two provided sequences. If unsuccessful, the child had reached their maximum level for that condition. Subsequently, the following condition was similarly explained and launched. To mitigate potential fatigue, which could lead to rejection of the games, the games were designed to be relatively short, with session durations adapted to the individual capacities of the children. A 5-minute break was incorporated before the onset of the third condition for the AR Corsi and AR Zoo games. Additional breaks were permitted if required by the child or deemed necessary by the therapist.

Each session encompassed three steps: (1) a presession assessment was conducted, comprising a physical and mental fatigability scale (Rating scale of Perceived Exertion for Children [RPE-C]); (2) the main session included a familiarization phase followed by the children’s engagement with one of the two AR games; and (3) a postsession evaluation was conducted, incorporating a usability questionnaire (System Usability Scale [SUS]), a UX questionnaire (AttrakDiff/MeCue), and a postsession fatigability questionnaire (RPE-C).

#### UX Assessment

The usability of the device, a subconcept of UX, was assessed by means of the SUS questionnaire [47]. This questionnaire is a validated tool commonly used in usability studies of new technologies [48], including AR with HoloLens 2 [49]. It consists of 10 statements. Children rate their agreement with each statement using a 5-point Likert scale with anchor points at either end (“Strongly Disagree” to “Strongly Agree”). The results of the SUS questionnaire help to calculate a standardized SUS score. Generally, a score above 85 is considered “excellent,” above 75 is “good,” and between 50 and 75 is “fair” or “acceptable.” A score below 50 indicates low user satisfaction [50].

The UX was assessed by means of the AttrakDiff questionnaire [51,52]. This tool consists of 28 items divided into four dimensions: pragmatic quality, hedonic quality (stimulation), hedonic quality (identity), and overall attractiveness. The 28 items are presented as pairs of contrasted semantic differentiators on a 7-point scale (from –3 to 3). The score for each dimension is analyzed separately. Scores close to the mean (within the 0 to 1 range) are considered standard. The AR device fulfills its purpose without negative impact, but improvements are possible to enhance attractiveness. Scores between 1 and 3 are considered positive points, while scores between –1 and –3 are considered negative points regarding the device, of which the UX was also evaluated using the emotional dimension of the MeCue UX scale [53]. Children rated eight statements (four positive emotions and four negative emotions) using a 7-point Likert scale with anchor points at either end (“Strongly Disagree” to “Strongly Agree”).

Secondary effects (mental and physical fatigue) were assessed using the RPE-C visual analog scales before and after the session [54]. Ratings range from 6 (no fatigue) to 20 (extreme fatigue). Pictograms in the form of smiley faces are used to assist children.

#### Performance Assessment

Performance was assessed by measuring the maximum level reached by the player for each variant of the two games. This approach enabled us to establish a baseline of performance among typically developing participants.

### Statistical Analysis

Based on the study by Balani and Tümler [49], with a theoretical SUS good score of 75, an effect size of 0.65, an α risk of 5%, a power of 90%, and a study exit mark-up of 15%, we determined that a sample size of 30 participants was required for this study.

Participant characteristics and performance outcomes were described using descriptive statistics. The SUS questionnaire data were analyzed using a normality test and descriptive statistics for each game. The AttrakDiff questionnaire data and the emotions item of the MeCue questionnaire were analyzed using descriptive statistics (mean or median value of each subscale) for each game, depending on normality. Depending on data distribution, a two-tailed independent samples *t* test or a Mann-Whitney test was used to determine whether or not there are significant differences between the two games. A mixed ANOVA was conducted to investigate the main effects of the “Time” (before and after the session) and “Game” (AR Corsi and AR Zoo) factors, as well as their interactions on physical and mental fatigue. Statistical analyses were conducted using RStudio (Posit Software, PBC) software. The level of statistical significance for all analyses was .05.

## Results

### Participants’ Score and Performance of AR Games

A total of 30 participants were included in our study. Among them, three participants were excluded due to incomplete questionnaire data. Data from 27 participants, with a distribution of 6 girls and 21 boys, were analyzed. The average age was 11.9 (SD 1.2) years.

**Figure 6:**
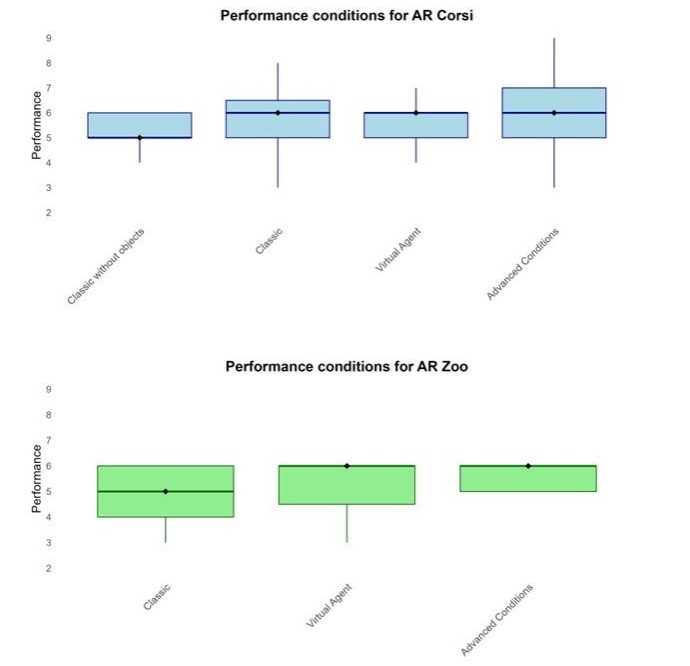
The performance results. Boxplots illustrate the distribution of performance scores for each condition and each game (AR Corsi and AR Zoo). Each plot includes the median (represented by the central line), with the lower quartile (Q1) and upper quartile (Q3) indicated by the edges of the box, as well as the extremes. The maximum level for the AR Corsi game is set at 9, while for the AR Zoo game, it is set at 6.

The mean effective gameplay durations for the sessions with the AR Corsi and the AR Zoo games were 35.7 (SD 6.1) and 42.9 (SD 8.7) minutes, respectively. The performance results for the AR Corsi and AR Zoo games are visualized in Figure 6, showing the median scores (Q1-Q3) across the different conditions for each game.

### UX Assessment

The UX results are presented in Table 1 (data tables in Multimedia Appendix 3).

Regarding usability, the mean scores for the Corsi and Zoo games were 79.9 (SD 11.4) and 76.3 (SD 12.1) out of 100, respectively, with no significant difference between the two games (Table 1). For the AR Corsi game, the mean scores for each scale of the AttrakDiff questionnaire were as follows: global attractiveness 1.7 (SD 0.7), pragmatic quality 1.2 (SD 0.8), and hedonic qualities “identity” and “stimulation” 0.6 (SD 0.5) and 1.1 (SD 0.7), respectively. Regarding the AR Zoo game, the mean scores for each scale of the AttrakDiff questionnaire were: global attractiveness 1.8 (SD 0.9), pragmatic quality 1.3 (SD 0.7), and hedonic qualities “identity” and “stimulation” 0.7 (SD 0.8) and 1.1 (SD 0.8), respectively. There was no significant difference between the two AR games for the four AttrakDiff variables (Table 1).

In the emotions module of the MeCue questionnaire, participants had an average score of 5.2 (SD 0.7) for the Corsi game and 5.3 (SD 0.8) for the Zoo game, both of them significantly higher than the theoretical scale value of 4 (*P*<.001). There was no significant difference between AR Corsi and AR Zoo (Table 1).

The results of the mixed ANOVA demonstrated a statistically significant main effect of the “Time” factor on physical and mental fatigue, with the respective *P* values of *P*=.02 and *P*=.002. In contrast, no significant difference was observed for the “Game” factor on physical and mental fatigue (*P*=.77 and *P*=.29). Furthermore, no statistically significant interaction was identified between the type of game and time on physical and mental fatigue scores (*P*=.54 and *P*=.28).

**Table 1:** Presentation of UX^a^ results and comparison between the games.

			AR Corsi, mean (SD)		AR Zoo, mean (SD)		Game comparison, *P* value
SUS^b^ (0 to 100)			79.9 (11.4)		76.3 (12.1)		.26
**AttrakDiff** (–3 to 3)							
	Overall attractiveness	1.7 (0.7)		1.8 (0.9)		.49	
	Identity	0.6 (0.5)		0.7 (0.8)		.66	
	Pragmatic quality	1.2 (0.8)		1.3 (0.7)		.85	
	Stimulation	1.1 (0.7)		1.1 (0.8)		.82	
MeCue (1 to 7)			5.3 (0.8)		5.4 (0.7)		.73

^a^UX: user experience.

^b^SUS: System Usability Scale.

## Discussion

### Principal Findings

This study introduces an innovative AR exergame designed for the cognitive-motor rehabilitation of children with CP or ABI. Following a co-design approach based on the MIDE framework, the objective was to test these games with typically developing children in view of gathering initial impressions on UX before offering the game to youth with CP or ABI.

Usability, a subset of UX, is considered a fundamental design criterion, particularly in the field of health technologies, where an adequate level of usability is not only expected but required [55]. In this study, the average SUS scores for the AR Corsi and AR Zoo games were 79.9 (SD 11.4) and 76.3 (SD 12.1) out of 100, respectively. According to the classification established by Bangor et al [50], these results correspond to a usability rating of “good,” which confirms our primary hypothesis regarding the ease of use of our AR exergames. These results are consistent with those obtained by Lauer et al [56], who likewise observed “good” usability (80/100) in their study on HoloLens 2 use. In their research, 47 elementary school children assessed positively the usability of an AR device using Microsoft HoloLens 2, with no reported cybersickness [56]. The absence of cybersickness, which has often been associated with VR, is a significant advantage of AR, particularly insofar as this phenomenon can markedly impair the UX in rehabilitation contexts [57].

We ensured that our exergames not only were user-friendly for typically developing children but also provided a positive UX. UX evaluation by means of the AttrakDiff questionnaire yielded favorable results, with scores ranging between 1 and 3 points for overall attractiveness, pragmatic quality, and stimulation for both the AR Corsi game and the AR Zoo game [51,52]. However, the hedonic quality dimension “identity” received more modest and neutral scores (mean 0.6, SD 0.5) for AR Corsi and (mean 0.7, SD 0.8) for AR Zoo, indicating an area with room for improvement. A similar observation was put forward by Le Roy et al [28] in a pilot study with 14 healthy adult participants, in which a score of 0.67 for hedonic quality (identity) was recorded for a VR device addressed to patients with depression. This result was attributed to the difficulty of their system to reflect the user’s identity in a context where the device was not intended for them. Similarly, in this study, we suggest that the neutral results for this variable could be due to the fact that these exergames, designed for children with CP or ABI, did not meet the expectations or preferences of typically developing children. These children may be less inclined to identify with rehabilitation games, which are quite different from the commercial games to which they are accustomed. It is likely that this dimension of UX would be rated more positively if evaluated by the target population.

Our exergame generated significant emotional engagement among typically developing youth. The results obtained with the MeCue questionnaire for the AR Corsi and AR Zoo games significantly exceeded the theoretical scale average set at 4, indicating the emergence of positive emotions. Consistent with the Mahlke model, these results suggest that UX plays a crucial role in eliciting positive emotional responses [24]. A pleasant UX increases the likelihood of evoking positive emotions in the user. Furthermore, as noted by Dirin and Laine [58], emotional engagement is key to promoting sustained use, particularly in AR mobile apps. Therefore, it will be important to evaluate this aspect with the target population in view of ensuring that the exergame fully meets their needs while remaining emotionally engaging and user-friendly, thereby contributing to the child’s sense of self-value and reinforcing their self-esteem.

While it is known that AR generally does not induce cybersickness, there is a gap in the literature regarding the evaluation of physical and mental fatigue caused by prolonged use of XR technologies among children and adolescents aged 10 to 16 years. We hypothesized that AR-based sessions would not result in a significant additional increase in physical or mental fatigue compared to presession evaluations. However, this study’s results reveal a significant effect of physical fatigue (*P*=.02) and mental fatigue (*P*=.002) between pre- and postsession measurements for both games, a finding contradicting our initial hypothesis.

Regarding observed physical fatigue, it is likely that the weight of the headset played a part. Three participants reported discomfort due to the excessive weight of the headset, leading to neck pain. This can be partially explained by the fact that both games require increased body rotations, especially as the difficulty level rises. Our performance results indicate that, on average, typically developing children achieve high maximum levels for each game condition. Belmonti et al [36] made an interesting observation in their study on children with CP using the MC. Short sequences (up to three or four tiles) do not necessarily require a specific strategy, and body rotations are limited. However, as sequences become longer and visual monitoring grows more complex, players need to adopt strategies involving more frequent body rotations to maintain their point of view, a phenomenon that was likely exacerbated in this study by the limited field of view of the Microsoft HoloLens 2. Depending on the strategy chosen, rotations may be more or less frequent, potentially explaining the neck pain associated with headset weight, which is intensified by excessive rotations in some children.

Regarding mental fatigue, even though no significant difference was observed between AR Zoo and AR Corsi, it is likely that the cognitive load induced by the different game variants is too excessive. It bears mentioning that the observations presented here remain hypothetical, as we did not objectively measure the executive functions involved in the various game conditions. For both games, AR Corsi and AR Zoo, we designed variants that simultaneously engage multiple executive functions. For AR Corsi, we followed approaches described in the literature, introducing a simplified version with a virtual agent focused on visuospatial memory, and a more complex version requiring concurrent use of mental inhibition. Similarly, AR Zoo involves multiple executive functions, with sequence completion requiring cognitively demanding tasks: the player must integrate visuospatial memory with planning, replanning, and mental inhibition. The literature suggests that simultaneous activation of several executive functions, such as planning and inhibition in spatial navigation tasks, often results in increased cognitive fatigue and declining performance [59-61]. This cognitive overload is particularly pronounced in contexts where multiple cognitive processes need to be managed concurrently, as is the case in AR Zoo and AR Corsi. Moreover, it is reasonable to assume that the addition of AR intensifies the cognitive effort required, particularly for mental rotation skills and visuospatial memory, due to the movement in 3D space that involves changing perspectives.

In our development, we aimed to design two AR games offering an optimal UX, ensuring no significant differences between them. Our secondary hypothesis was confirmed, showing that typically developing children evaluated both games similarly in terms of UX. A key factor explaining this outcome is adherence to the MIDE model [21] during the exergame design, making sure that the games met user needs and expectations. Additionally, we built both games on a similar core loop, making it easier for young players to understand and engage. The literature emphasizes that a game’s primary loop is essential for maintaining motivation and engagement, especially in therapeutic contexts [25]. By providing a consistent foundation while introducing variants to target different executive functions and adjusting difficulty, we made sure that both games remained equally engaging and replayable. This approach could serve as a foundation for future development of pediatric cognitive-motor rehabilitation games.

### Limitations

This study has several limitations. First, the measurement tools used are based on scales and questionnaires, which although validated, remain inherently subjective. Additionally, some children’s understanding of certain questionnaires proved challenging, requiring rephrasing of certain terms, which may have influenced the results.

Another limitation is related to temporal bias, as not all of the sessions were conducted at the same time of day. Some participants played one exergame in the morning and another in the afternoon on different days. Moreover, participants continued their daily activities (physical, manual, etc) within a youth club or leisure center, which might have affected their level of fatigue. This temporal bias could introduce variations in the results, particularly regarding mental and cognitive fatigue, as factors such as attention and cognitive performance fluctuate throughout the day.

A final limitation concerns the use of the Microsoft HoloLens 2 headset. Some participants reported discomfort regarding the headset’s field of view, which they felt was limited. While the second version of the HoloLens 2 offers a field of view expanded by 18 degrees compared to the first version, this limitation had been noted in a previous study involving participants with CP using the first version. To ensure the flexibility of our exergames, we designed them to be easily transferable to other AR devices. In future studies, it would be beneficial to test our exergames on headsets with a wider field of view, which could potentially enhance the UX.

### Conclusions

This pilot study demonstrated that our exergame, including the AR Corsi and AR Zoo games, is not only easy to use but also well-received by typically developing youth, eliciting positive emotions. Although some children experienced physical or mental fatigue, the positive evaluation of the UX with this population represents an essential preliminary step in our codevelopment process, particularly regarding the verification of game content and mechanisms. With these methodological aspects having been validated, it is henceforth possible to expand the use of this exergame to children with CP or ABI.

After validating the mechanisms of our games and observing positive UX among typically developing youth, we can now proceed to offer our exergame to young individuals with CP or ABI. In line with the principles of the MIDE design framework [21], it will be crucial in a similar study to evaluate the UX of our exergame in this population. Following this second study, potential modifications may be made to our games based on the performance outcomes and feedback from children with CP and ABI.

In future studies, we plan to incorporate tools such as neuropsychological assessments or electroencephalography measurements to objectively evaluate the neural networks involved and to deepen our understanding of the cognitive processes engaged by the different conditions of our games. To explore the link with executive functions, we will conduct correlations with neuropsychological test batteries, similar to the study by Kronovsek et al [62].

## Appendix

Multimedia Appendix 1 AR Corsi game video.

Multimedia Appendix 2 AR Zoo game video.

Multimedia Appendix 3 User experience data tables.

## Abbreviations

ABI: acquired brain injury
AR: augmented reality
CP: cerebral palsy
MC: magic carpet
MIDE: Multidisciplinary Iterative Design of Exergame
RPE-C: Rating scale of Perceived Exertion for Children
SUS: System Usability Scale
UX: user experience
VR: virtual reality
XR: extended reality
