# Supplementary material for: Augmented Reality Exergames for Upcoming Cognitive-Motor Rehabilitation: User-Centered Design Approach and User Experience of Healthy Children
Source: JMIR Rehabil Assist Technol. 2025 Feb 19;12:e69205. doi: 10.2196/69205 (PMC11888016; doi:10.2196/69205)
Supplement: Multimedia Appendix 3 [file rehab_v12i1e69205_app3.pdf]

UX Data Table for AR CORSI

Participants	Physical fatigue pre	Mental fatigue pre	SUS	Pragmatic quality	Stimulation	Identity	Overall attractiveness	MeCue	Physical fatigue post	Mental fatigue post
1	8	6	72,5	1,14	0,71	0,86	0,86	5,25	8	6
2	6	8	92,5	1,57	0,57	0,29	1,14	4,5	6	10
3	8	8	80	0,57	2,14	1,57	1,86	4,75	9	7
4	10	9	67,5	1,71	-0,14	0,57	1,57	5,75	7	7
5	7	9	60	0,43	0,71	0,43	0,71	3,87	8	10
6	8	6	82,5	1,71	1,29	0,57	1,71	4,5	8	7
7	14	11	57,5	0,86	-0,43	0	1,14	5,75	14	10
8	6	7	90	2,29	1,14	0,86	2,43	5,13	6	8
9	8	7	67,5	-0,29	1,43	-0,29	1,71	4,38	9	10
10	6	7	70	0,14	0,71	0,57	1,43	4,88	6	7
11	8	6	92,5	0,71	2,29	0,14	2,43	6	8	7
12	6	6	100	2,14	0,71	0,57	2,71	6	6	6
13	8	7	70	1,14	0,57	0,57	0,86	4,75	10	8
14	10	6	82,5	1,29	1	0,86	1,57	5,25	11	7
15	10	11	72,5	0,43	0,71	1,14	1,43	5,75	11	12
16	6	7	95	1,14	1,57	0,71	1,14	5,13	10	9
17	7	8	82,5	1,29	2	1,29	1,71	6,63	7	7
18	6	7	85	1,43	1,71	0,71	1,86	5,25	7	7
19	7	7	80	2,86	1,86	0,28	2,43	6,13	8	7
20	7	8	80	1,14	1,29	1,14	1,29	4,62	7	9
21	7	11	62,5	0,71	0,71	0,71	1,29	5,25	8	10
22	6	9	92,5	1,71	1,29	0,57	2,43	6,25	6	7
23	6	6	87,5	2,43	2,43	0,86	2,14	4,63	6	7
24	6	6	80	1,29	1,71	1,29	2,71	6,88	6	6
25	7	8	80	-0,14	0	-0,43	0,14	4,38	7	8
26	8	7	80	2,14	0,86	0	2,29	6	9	8
27	7	7	95	1,57	1,86	1,57	2,29	6	7	7

UX Data Table for AR Zoo

Participants	Physical fatigue pre	Mental fatigue pre	SUS	Pragmatic quality	Stimulation	Identity	Overall attractiveness	MeCue	Physical fatigue post	Mental fatigue post
1	6	7	72,5	0,71	0,57	1,14	1	4,5	6	7
2	6	6	92,5	1,43	0,14	1	2,57	5	6	6
3	9	7	77,5	0,57	1,29	1,14	2	5	9	8
4	8	6	82,5	1,86	0,14	0	1,71	6	6	6
5	7	8	62,5	0,71	1	0,57	0,71	3,87	9	9
6	9	7	80	1,29	1,43	0,29	1,71	4,87	9	7
7	10	8	62,5	1,71	2	1,57	2,43	5,88	8	7
8	6	6	97,5	1,86	1	2,57	2,86	6	6	6
9	8	6	50	-0,14	0,43	-0,43	0,86	4	9	10
10	6	9	70	0,71	1,43	0,43	2	6,25	6	10
11	8	6	77,5	0,43	2,71	0,14	2,71	6,13	9	7
12	8	6	87,5	1,86	1,57	1,43	2,43	6,38	6	6
13	8	6	65	1,14	-0,14	1,43	1,29	4,75	9	8
14	9	7	82,5	1,86	1,14	0,57	0,86	4,88	10	8
15	12	10	57,5	1	1	1,43	1,14	6	12	11
16	7	8	95	0,57	0,14	0	0,57	5,13	10	10
17	7	8	75	0,71	1,71	0,43	3	5,38	7	7
18	7	6	85	2	2,14	1	2	6	7	7
19	7	6	77,5	2,43	2	0,86	2,57	5,13	8	8
20	7	7	72,5	1,29	1,43	1,57	1,14	4,75	8	9
21	8	10	55	1,29	0	0,57	1,14	5	9	10
22	6	7	87,5	1,71	1,14	0	2,57	6,38	6	7
23	6	6	80	2,14	1,29	0,29	2,57	5,5	6	7
24	6	6	85	0,86	0	1,14	2,43	6,13	6	6
25	8	8	67,5	-0,14	0,29	-0,86	-0,43	4,63	9	8
26	6	6	80	2,14	1,71	-0,43	3	6	6	6
27	7	7	82,5	2,43	1,86	1,71	2,43	6	7	7
